# Risk evaluation of denosumab and zoledronic acid for medication-related osteonecrosis of the jaw in patients with bone metastases: a propensity score–matched analysis

**DOI:** 10.1007/s00520-021-06634-7

**Published:** 2021-11-05

**Authors:** Hiroaki Ikesue, Kohei Doi, Mayu Morimoto, Masaki Hirabatake, Nobuyuki Muroi, Shinsuke Yamamoto, Toshihiko Takenobu, Tohru Hashida

**Affiliations:** 1 Department of Pharmacy, Kobe City Medical Center General Hospital, 2-1-1 Minatojima-Minamimachi, Chuo-ku, Kobe, Hyogo 650-0047 Japan; 2 Department of Oral and Maxillofacial Surgery, Kobe City Medical Center General Hospital, 2-1-1 Minatojima-Minamimachi, Chuo-ku, Kobe, Hyogo 650-0047 Japan

**Keywords:** Denosumab, Zoledronic acid, Osteonecrosis of the jaw, Risk factor

## Abstract

**Purpose:**

This study evaluated the risk of medication-related osteonecrosis of the jaw (MRONJ) in patients with cancer who received denosumab or zoledronic acid (ZA) for treating bone metastasis.

**Methods:**

The medical records of patients were retrospectively reviewed. Patients who did not undergo a dental examination at baseline were excluded. The primary endpoint was a comparison of the risk of developing MRONJ between the denosumab and ZA groups. Propensity score matching was used to control for baseline differences between patient characteristics and compare outcomes for both groups.

**Results:**

Among the 799 patients enrolled, 58 (7.3%) developed MRONJ. The incidence of MRONJ was significantly higher in the denosumab group than in the ZA group (9.6% [39/406] vs. 4.8% [19/393], *p* = 0.009). Multivariate Cox proportional hazards regression analysis revealed that denosumab treatment (hazard ratio [HR], 2.89; 95% confidence interval [CI], 1.65–5.25; *p* < 0.001) and tooth extraction after starting ZA or denosumab (HR, 4.26; 95% CI, 2.38–7.44; *p* < 0.001) were significant risk factors for MRONJ. Propensity score–matched analysis confirmed that the risk of developing MRONJ was significantly higher in the denosumab group than in the ZA group (HR, 2.34; 95% CI, 1.17–5.01; *p* = 0.016).

**Conclusion:**

The results of this study suggest that denosumab poses a significant risk for developing MRONJ in patients treated for bone metastasis, and thus these patients require close monitoring.

## Introduction

Bone metastasis is commonly found in patients with advanced cancers; it leads to clinically important complications, such as pain, hypercalcemia, spinal cord compression, and fractures [1, 2]. Skeletal-related events remarkably decrease the quality of life of patients with bone metastasis. Since recent progress in cancer treatments has prolonged the survival of patients with advanced cancer, the prevalence of bone metastasis in cancer patients has inevitably increased, accompanied by a further increase in the significance of corresponding treatments [3–12].

Zoledronic acid (ZA) is a potent bisphosphonate (BP) that has a high affinity for hydroxyapatite; it is internalized by osteoclasts during resorption, leading to inhibition of osteoclast function [13]. Denosumab is a fully humanized monoclonal antibody against nuclear factor-kappa B (NFκB) ligand (RANKL) [14]. Both ZA and denosumab demonstrated efficacy in preventing skeletal complications in patients with bone metastasis secondary to solid tumors or osteolytic lesions of multiple myeloma. Despite the usefulness of bone-modifying agents (BMAs), significant safety concerns including hypocalcemia [15] and medication-related osteonecrosis of the jaw (MRONJ) have been reported as severe side effects associated with their use [16]. MRONJ causes severe pain and markedly reduces patient quality of life; therefore, collaborative management with healthcare providers, including dentists, is recommended. This approach will make it possible to conduct appropriate monitoring, assessment, and treatment in early-stage MRONJ [17–20]. Thus, it is necessary to accumulate more information on the risk factors for osteonecrosis of the jaw.

Several risk factors and comorbid conditions that contribute to the development of MRONJ include BMAs, duration of therapy, dental extraction and other oral surgical procedures, periodontal disease, oral health status, tobacco use, angiogenesis inhibitors, corticosteroids, diabetes, and increasing age [17–19, 21, 22]. Reported risk evaluations for BMAs remain controversial. Although a recent meta-analysis based on the results from randomized controlled trials reported that denosumab was associated with a significantly higher risk of developing MRONJ than ZA [23], this significant difference between the two drugs was not shown in other meta-analyses [24–27]. Furthermore, in real-world clinical practice, preventing MRONJ is more complex. This complexity is due to the treatments, including those for various patients with more risk factors, such as older patients, unscheduled dental treatments, diabetic complications, and the concomitant use of medications that increase the risk of MRONJ. However, there is little information available regarding risk evaluation comparing BMAs in real-world clinical practice. Moreover, no available studies have compared the risk of MRONJ between denosumab and ZA based on adjusted baseline patient characteristics. In this study, to reduce the impact of potential bias in an observational study, we conducted a propensity score–matched analysis in a clinical setting to compare the risk of developing MRONJ between patients with cancer bone metastasis treated with the BMAs denosumab or ZA.

## Methods

### Study design, setting, and patient population

This retrospective observational study was conducted in accordance with the Declaration of Helsinki. The study protocol was approved by the Internal Review Board of the Kobe City Medical Center General Hospital (approval number: k191010). Due to the retrospective nature of this work, informed consent was waived for the individual participants included in the study in accordance with the ethical standards of the Internal Review Board of the Kobe City Medical Center General Hospital, and the research plan was published on the homepage of the hospital in accordance with the guaranteed opt-out opportunity. Adult patients were eligible if they were diagnosed with cancer, presented with at least one bone metastasis or osteolytic lesion, and started denosumab or ZA treatment at Kobe City Medical Center General Hospital after dental examinations by dentists between July 1, 2011, and October 31, 2019. The following comprised the exclusion criteria: lack of dental examination before beginning denosumab or ZA treatment, use of ZA for hypercalcemia treatment, could not be followed up 1 month after the start of BMA treatment, or history of radiation therapy for the jaw.

### Bone metastasis treatment procedure

When needed and following dental examination, patients underwent dental procedures (such as tooth extraction) to minimize the risk of MRONJ development prior to initiating BMA treatment. All patients were administered 120 mg denosumab subcutaneously every 4 weeks or 4 mg ZA intravenously every 3–4 weeks. Patients with decreased kidney function (creatinine clearance ≤ 60 mL/min) were administered a reduced ZA dose based on their creatinine clearance, as recommended by the manufacturer. We divided the study subjects into two groups: patients who received denosumab (denosumab group) and patients who received ZA (ZA group).

### Data collection and assessment

All data were collected from the electronic medical record system. We evaluated information regarding sex, age, weight, cancer type, comorbidities, tooth extraction before and after starting BMA treatment, concomitant medications, type of BMA, number of treatment courses, and outcomes of treatment for MRONJ. To minimize potential bias in evaluating factors associated with MRONJ development, study participants were limited to those examined by dentists before initiating BMA treatments, because poor oral health status has been reported as a remarkable risk factor for MRONJ [17–20]. Moreover, it was recommended that all patients routinely visit dental clinics after the initiation of BMA treatment. If the patients were considering invasive dental procedures, such as tooth extraction, after commencing BMA treatment, they were asked to consult with dentists in our hospital. After starting BMA treatment, patients who complained of dental symptoms, including pain or oral discomfort, consulted a dentist according to the request of the attending physician. In unavoidable situations, including accidental root fracture or acute exacerbation of periodontal disease, tooth extraction was performed. MRONJ was diagnosed by dentists in our hospital based on clinical and radiographic findings, according to the criteria stated in the American Association of Oral and Maxillofacial Surgeons (AAOMS) position paper [20, 28], and the cut-off date when the patients were diagnosed as MRONJ was December 31, 2019. Since switching from ZA to denosumab can increase the risk for developing MRONJ, for patients who received ZA followed by denosumab, the cut-off date was the day of switching to denosumab treatment. Similarly, in patients who received denosumab followed by ZA, the cut-off date was the day of switching to ZA. Thus, all MRONJ cases in this study were assessed between they received only denosumab or ZA. The primary endpoint was a comparison of the risk of developing MRONJ between the denosumab and the ZA groups, whereas secondary endpoints included the risk factors for developing MRONJ and the relationship between risk factors and the time to MRONJ development.

### Statistical analysis

Categorical data are presented as numbers (percentages) and were compared between groups using the chi-square test. Continuous data are presented as medians (interquartile ranges), and the Mann–Whitney *U* test was used to compare the groups. The Cox proportional hazards regression model was employed to analyze the associated factors for developing MRONJ. Variables with a *p*-value < 0.05 in the univariate analyses were applied to the multivariate analysis. The cumulative incidences of MRONJ were described by the Kaplan–Meier method, and the differences of the time to development of MRONJ were compared with the log-rank test.

To adjust for the other baseline factors, a sensitivity analysis was conducted by propensity score matching. We estimated the propensity score by modeling the probability of being in the ZA group versus the denosumab group. The following variables were included in the regression model: sex, age, cancer type, tooth extraction before starting BMA treatment, comorbidity with diabetes, concomitant use of antiangiogenic agents, concomitant use of corticosteroids, and tooth extraction after starting BMA treatment. To reduce bias with these potential confounding factors, 1:1 matching (without replacement) in the two treatment groups was achieved using the nearest neighbor method with a 0.20-width caliper of standard deviation of the logit of propensity scores. The matched data were analyzed to confirm the robustness of the primary analysis results. We used JMP add-in package version 13.2.1 (SAS Institute Inc., Cary, NC, USA) for all statistical analyses, and two-tailed *p*-values < 0.05 indicated statistical significance.

## Results

### Patient characteristics

**Fig. 1:**
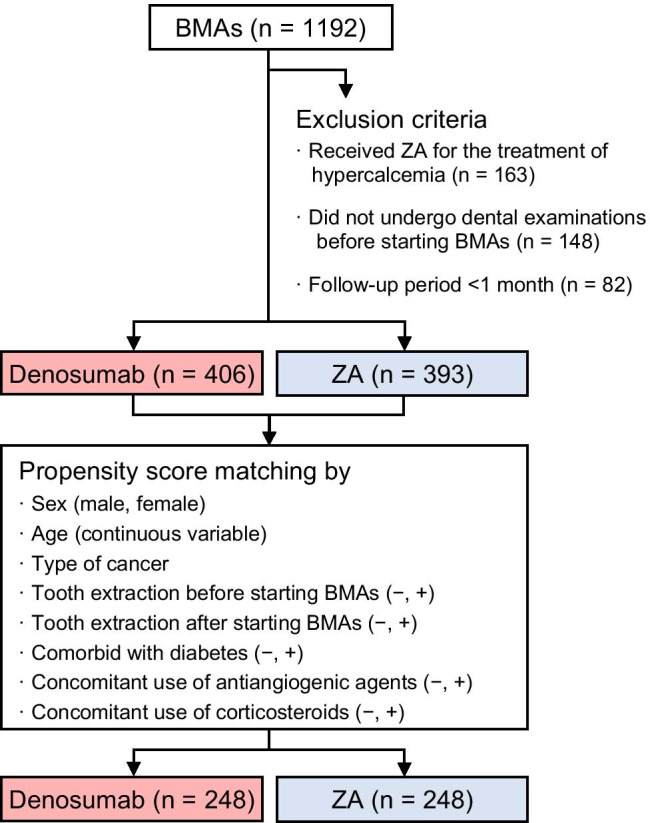
Study diagram. BMA, bone-modifying agent; ZA, zoledronic acid

Between July 2011 and October 2019, 1192 consecutive adult patients with cancer bone metastasis were administered denosumab or ZA (Fig. 1). Among them, 799 patients (406 in the denosumab group and 393 in the ZA group) met the inclusion criteria, and their characteristics are summarized in Table 1. Three hundred and ninety-three patients were excluded because they received ZA for the treatment of hypercalcemia (*n* = 163), lacked dental examinations before starting denosumab or ZA treatment (*n* = 148), or could not be followed up for 1 month after the start of BMA treatment (*n* = 82). Before propensity score matching, the proportion of male patients was significantly higher in the denosumab group than in the ZA group. The proportions of patients with lung cancer or prostate cancer were higher in the denosumab group, whereas those with multiple myeloma were higher in the ZA group. The proportion of patients who received concomitant corticosteroids was significantly higher in the denosumab group than in the ZA group. The incidence of MRONJ in all study subjects was 7.3% (58/799) and was significantly higher in the denosumab group than in the ZA group (9.6% [39/406] vs. 4.8% [19/393], *p* = 0.009]. Stage 0, 1, 2, and 3 MRONJ were developed in 1, 9, 27, and 2 patients in the denosumab group, and in 0, 4, 14, and 1 patients in the ZA group, respectively. After propensity score matching, patient characteristics were well balanced based on all measured variables (Table 1). In the propensity score–matched cohort, the incidence of MRONJ in the denosumab group was also significantly higher than that in the ZA group (9.7% [24/248] vs. 4.8% [12/248], *p* = 0.038).

**Table 1:** Patient characteristics before and after propensity score matching

Characteristics	Before propensity score matching			After propensity score matching		
	Denosumab (*n* = 406)	ZA (*n* = 393)	*p*-value	Denosumab (*n* = 248)	ZA (*n* = 248)	*p*-value
Male sex, *n* (%)	225 (55.4%)	188 (47.8%)	0.032	127 (51.2%)	125 (50.4%)	0.857
Age (years), median (IQR)	69 (61–75)	67 (60–75)	0.153	67 (60–74)	67 (59–74)	0.667
Type of cancer, *n* (%)						
Lung cancer	183 (45.1%)	98 (24.9%)	< 0.001	105 (42.3%)	98 (39.5%)	0.980
Breast cancer	86 (21.2%)	63 (16.0%)		59 (23.8%)	63 (25.4%)	
Multiple myeloma	6 (1.5%)	123 (31.3%)		6 (2.4%)	6 (2.4%)	
Prostate cancer	86 (21.2%)	37 (9.4%)		36 (14.5%)	37 (14.9%)	
Others	45 (11.1%)	72 (18.3%)		42 (16.9%)	44 (17.7%)	
Tooth extraction before starting BMAs, *n* (%)	92 (22.8%)	82 (20.9%)	0.503	51 (20.6%)	49 (19.8%)	0.823
Comorbid with diabetes, *n* (%)	76 (18.7%)	69 (17.6%)	0.670	37 (14.9%)	37 (14.9%)	1.000
Concomitant medication, *n* (%)						
Antiangiogenic agents^a^	102 (25.1%)	84 (21.4%)	0.210	75 (30.2%)	70 (28.2%)	0.622
Corticosteroids	58 (14.3%)	37 (9.4%)	0.033	19 (7.7%)	24 (9.7%)	0.425
Tooth extraction after starting BMAs, *n* (%)	36 (8.9%)	34 (8.7%)	0.888	21 (8.5%)	21 (8.5%)	1.000
Number of treatment courses, median (IQR)	8 (3–17)	7 (3–19)	0.637	7 (3–15)	6 (2–17)	0.340

For continuous values, data are presented as the median (interquartile range [*IQR*])

^a^Includes axitinib, bevacizumab, everolimus, lenvatinib, pazopanib, ramucirumab, regorafenib, sorafenib, sunitinib, and temsirolimus

*BMA*, bone-modifying agent; *MRONJ*, medication-related osteonecrosis of the jaw, *ZA*, zoledronic acid

### Risk factors for MRONJ

Univariate analysis showed that treatment with denosumab (hazard ratio [HR], 2.65; 95% confidence interval [CI], 1.53–4.77; *p* < 0.001), tooth extraction after starting BMAs (HR, 4.84; 95% CI, 2.78–8.24; *p* < 0.001), tooth extraction before starting BMAs (HR, 2.33; 95% CI, 1.37–3.93; *p* = 0.002), comorbidity with diabetes (HR, 0.37; 95% CI, 0.13–0.84; *p* = 0.014), and concomitant use of antiangiogenic agents (HR, 1.83; 95% CI, 1.06–3.10; *p* = 0.031) were significantly associated with the development of MRONJ in cancer patients who received BMA treatment (Table 2). Subsequent multivariate Cox proportional hazards model analysis also showed that denosumab treatment (HR, 2.89; 95% CI, 1.65–5.25; *p* < 0.001) and tooth extraction after starting BMAs (HR, 4.26; 95% CI, 2.38–7.44; *p* < 0.001) were significantly associated with a risk of developing MRONJ in cancer patients who received BMA treatment.

**Table 2:** Cox proportional hazards model for medication-related osteonecrosis of the jaw in patients who received denosumab or zoledronic acid for bone metastasis

Variables	Univariate analysis		Multivariate analysis		PS-matched analysis	
	HR (95% CI)	*p*-value	HR (95% CI)	*p*-value	HR (95% CI)	*p*-value
Bone-modifying agents						
ZA (control)	1.00	–	1.00	–	1.00	–
Denosumab	2.65 (1.53–4.77)	< 0.001	2.89 (1.65–5.25)	< 0.001	2.34 (1.17–5.01)	0.016
Tooth extraction after starting BMAs	4.84 (2.78–8.24)	< 0.001	4.26 (2.38–7.44)	< 0.001	–	–
Tooth extraction before starting BMAs	2.33 (1.37–3.93)	0.002	1.70 (0.98–2.92)	0.061	–	–
Diabetes	0.37 (0.13–0.84)	0.014	0.45 (0.15–1.04)	0.062	–	–
Concomitant use of antiangiogenic agents^a^	1.83 (1.06–3.10)	0.031	1.57 (0.90–2.68)	0.107	–	–
Male sex	1.21 (0.72–2.04)	0.482	N/A		–	–
Concomitant use of corticosteroids	0.98 (0.51–1.78)	0.959	N/A		–	–
Age (years)	1.02 (0.99–1.04)	0.178	N/A		–	–

N/A indicates that the covariate was not included in the model because it was not significant in univariate analyses

^a^Includes axitinib, bevacizumab, everolimus, lenvatinib, pazopanib, ramucirumab, regorafenib, sorafenib, sunitinib, and temsirolimus

*BMA*, bone-modifying agent; *CI*, confidence interval; *HR*, hazard ratio; *PS*, propensity score; *ZA*, zoledronic acid

**Fig. 2:**
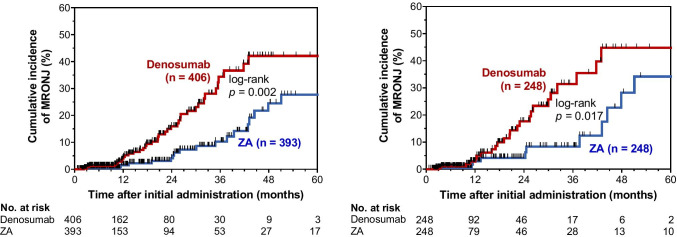
Cumulative incidence of MRONJ in patients receiving denosumab or ZA for bone metastasis. The cumulative incidences of developing MRONJ were compared between the denosumab and ZA groups (**a**) before and (**b**) after propensity score matching of the cohort. MRONJ, medication-related osteonecrosis of the jaw; ZA, zoledronic acid

We also performed propensity score matching to balance patient characteristics between the denosumab and ZA groups. There were no significant differences in the factors between the two groups (Table 1). In the propensity score–matched cohorts, the risk of developing MRONJ was significantly higher in the denosumab group than in the ZA group (HR, 2.34; 95% CI, 1.17–5.01; *p* = 0.016; Table 2). Kaplan–Meier curves of the time to MRONJ onset in the denosumab and ZA groups are shown in Fig. 2. The cumulative incidences of MRONJ in the denosumab group were significantly higher than those in the ZA group in both cohorts (*p* = 0.002, Fig. 2a) and in the propensity score–matched cohort (*p* = 0.017, Fig. 2b).

## Discussion

This is the first study showing that denosumab treatment can significantly increase the risk of developing MRONJ, when compared to ZA, among patients who received BMA treatment for bone metastasis in a real-world clinical practice setting using a propensity score–matched analysis. Tooth extraction after starting BMA treatment was also a significant risk factor. The incidences of MRONJ in the present study were 9.6% and 4.8% in the denosumab and ZA groups, respectively. The reported incidence of MRONJ is 1–17% [25, 27, 29–42], and the incidences of MRONJ in our study were within this range for both groups. In a combined analysis of three phase III randomized control trials, the incidences of MRONJ were low in patients receiving both denosumab (1.8%; *n* = 52/2862) and ZA (1.3%; *n* = 37/2861) [25]. Another randomized control trial also reported that the incidences of MRONJ were low in patients with multiple myeloma receiving both denosumab (4.1%; *n* = 35/850) and ZA (2.8%; *n* = 24/852) [37]. In contrast, in retrospective observational studies, the reported incidences of MRONJ were found to vary from 2 to 17% [29–31, 38–42] and were relatively higher than those in randomized controlled trials. In randomized controlled trials, the study protocol specified that all participants underwent scheduled periodic dental examinations (e.g., at baseline and every 6 months thereafter) [25, 27, 32–34, 36, 37]. On the other hand, in the real-word clinical practice setting used in our study, although all subjects underwent dental examination before the initiation of BMA treatment, the vast majority of them did not receive scheduled periodic dental examinations after the initiation of BMA treatment. If the patients complained of dental symptoms, the attending physicians consulted the dentists. Because scheduled dental examination can reduce the risk of developing MRONJ [17–19, 31, 35, 41–43], this discordance between randomized control trials and observational studies in real-world clinical practice might affect the risk of MRONJ.

This study revealed via multivariate analysis that denosumab treatment was associated with a significantly higher risk of developing MRONJ than ZA treatment. Moreover, this result was also clearly confirmed by propensity score–matched analysis. The higher risk for developing MRONJ with denosumab than with ZA was consistent with some previous real-world data [40–43]. Previous randomized controlled trials reported that the incidence of MRONJ in patients treated with denosumab was not significantly different from that in patients treated with ZA, although it tended to be higher in the former [25, 27, 32–34, 36, 37]. One potential explanation for this discordance might be due to scheduled dental examination, which we discussed previously. However, when investigating the risk of MRONJ in retrospective observational studies, several biases, such as dental examinations before BMA treatments and dental interventions after starting BMA treatments, should be considered. As such, to reduce the potential bias of patient characteristics associated with the development of MRONJ, we limited the study participants to those examined by dentists before starting BMA treatment. In addition, we performed propensity score matching to control for and reduce selection bias in the denosumab and ZA groups. Recent study and the European Task Force on MRONJ pointed out that alveolar bone histological necroses were observed prior to teeth extractions in patients who were treated with BMAs [44, 45]. Although the results of multivariate analysis in our study showed that tooth extraction after the start of BMA treatment as a significant risk factor, it is important to note that it is highly possible that MRONJ have developed before tooth extraction in our study subjects. Unfortunately, we could not obtain data on alveolar bone necroses prior to teeth extractions in our study subjects because they have received dental procedures at dental clinics rather than our hospital. Taken together, we believe that educating patients and routine periodic dental examinations are essential to reduce the risk of developing MRONJ.

The higher incidence of denosumab-associated osteonecrosis of the jaw seems to reflect its superior effect on bone resorption compared to that of ZA [26, 32, 33]. In clinical practice, careful monitoring of developing MRONJ is essential in all patients receiving denosumab and/or ZA, even if the risk of developing MRONJ in this study was significantly lower than those in the denosumab group. Recently, we reported that MRONJ caused by denosumab resolves faster than that caused by ZA [40]. Bacci et al. reported the importance of appropriate dental visits and treatments in reducing the risk of MRONJ [46]. We believe that diagnosing MRONJ at an earlier stage through appropriate monitoring and multidisciplinary collaborative work with healthcare providers is essential for BMA treatment [47].

We also revealed that tooth extraction after starting BMA treatment was significantly increased by multivariate analysis, which was consistent with the findings of previous reports [19, 21, 25]. In contrast, tooth extractions performed before starting BMAs tended to increase the risk of developing MRONJ in this study, although this increase was not significant. Poor oral health status is known to be a significant risk factor [17–20], and our results likely support the notion that in unavoidable cases, tooth extraction before starting BMAs is a meaningful intervention to reduce the risk of developing MRONJ. However, in this retrospective study, we could not obtain convincing data related to oral health status before starting BMAs. Further studies are required to confirm these results. Since tooth extraction before starting BMAs significantly increased the risk of developing MRONJ in the univariate analysis, early dental consultation should be considered after patients are diagnosed with cancer.

This study has some limitations. First, oral health status, such as periodontal disease and dental caries, was not fully investigated. To reduce the effect of these factors, we limited the study participants to those examined by dentists before starting BMA treatment. Second, we did not evaluate the effect of other risk factors, such as dental prosthesis and tobacco use [17–20]. Thirdly, we also could not evaluate data on interval between the initiation of BMA treatments and the date when the patients receive dental evaluation. Similarly, detailed information on dental procedures, and how long time has elapsed between dental extractions and the start of bone-modifying agents are important. In this study, however, we could not obtain exact information, because some patients have visited other dental clinics. Despite our best efforts to obtain clinical information, we were not able to collect all of these data within this retrospective study design. However, to our knowledge, these missing data should have similar impacts among the groups. Lastly, since the patients who complained of dental symptoms consulted a dentist following the request of the attending physicians, dental examinations might be performed when patients had developed oral symptoms, and mild cases of MRONJ might have been underdiagnosed. Despite these limitations, this real-world observational study clearly revealed that the risk of developing MRONJ was significantly higher in advanced cancer patients treated with denosumab than in those treated with ZA.

In conclusion, the results of this study suggest that denosumab significantly increases the risk of developing MRONJ compared to ZA in cancer patients undergoing treatment for bone metastasis. Tooth extraction after starting BMA treatment is also significantly associated with developing MRONJ. Taken together, these patients require close monitoring in a clinical setting.

## Data Availability

The data underlying this study are contained within the article.
